# Supplementary material for: Identification of photoperiod-induced specific miRNAs in the adrenal glands of Sunite sheep (Ovis aries)
Source: Front Vet Sci. 2022 Jul 22;9:888207. doi: 10.3389/fvets.2022.888207 (PMC9354845; doi:10.3389/fvets.2022.888207)
Supplement: Supplementary file 1 [file Data_Sheet_1.ZIP › Supplementary file/miRDeep2 file.zip!/miRDeep2 file/Novel_Structure/LP42A2/Novel_96.pdf]

Provisional ID	:	NC_019484.2_37517
Score total	:	2.1
Score for star read(s)	:	-1.3
Score for read counts	:	0
Score for mfe	:	1.8
Score for randfold	:	1.6
Score for cons. seed	:	
Total read count	:	41
Mature read count	:	40
Loop read count	:	0
Star read count	:	1

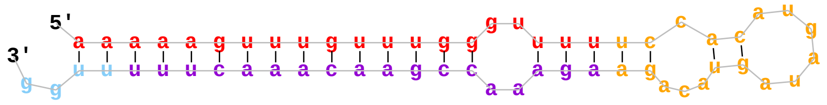

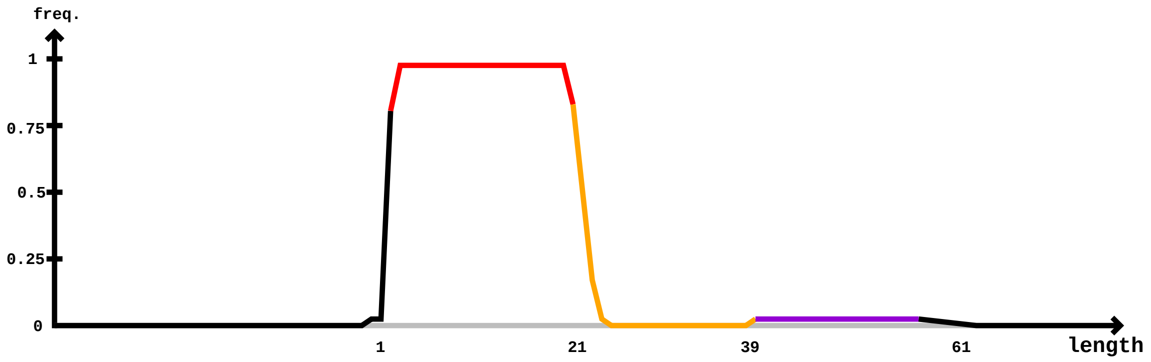

Mature Star

```
5'- agggugaggaacucgguacuauugggguuggcaaaaaaguuuguuuggguuuuuccacaugauaguacagaagaaaccgaacaaacuuuuuggcuaauccaauagcagc   -3'   obs
    agggugaggaacucgguacuauugggguuggcaaaaaaguuuguuuggguuuuuccacaugauaguacagaagaaaccgaacaaacuuuuuggcuaauccaauagcagc         exp
    .((((.....))))....((((((((.(((((.(((((((((((((((..(((((.((......))...)))))..))))))))))))))).)))))))))))))....   reads   mm   sample
    ...............................caaaaaaguuuguuuggguuA.........................................................   1       1    seq
    .................................aaaaaguuCguuuggguuu.........................................................   5       1    seq
    .................................aaaaaguuCguuuggguuuu........................................................   24      1    seq
    .................................aaaaaguuGguuuggguuuu........................................................   1       1    seq
    .................................aaaaaguuuguuuggguuuuu.......................................................   2       0    seq
    ..................................aaaGguuuguuuggguuuuuc......................................................   3       1    seq
    ..................................aCaaguuuguuuggguuuuuc......................................................   1       1    seq
    ..................................aaaaguuuguuuggguuuuuc......................................................   2       0    seq
    ..................................aaaaguuuguuuggguuuuucc.....................................................   1       0    seq
    .......................................................................agaaaccgaacaaacuuu....................   1       0    seq
```
